# Supplementary material for: Microenvironmental Snail1-induced immunosuppression promotes melanoma growth
Source: Oncogene. 2023 Jul 29;42(36):2659–72. doi: 10.1038/s41388-023-02793-5 (PMC10473961; doi:10.1038/s41388-023-02793-5)

SUPPLEMENTARY FIGURES AND LEGENDS

Supplementary Figure 1 (Related to Figure 1)

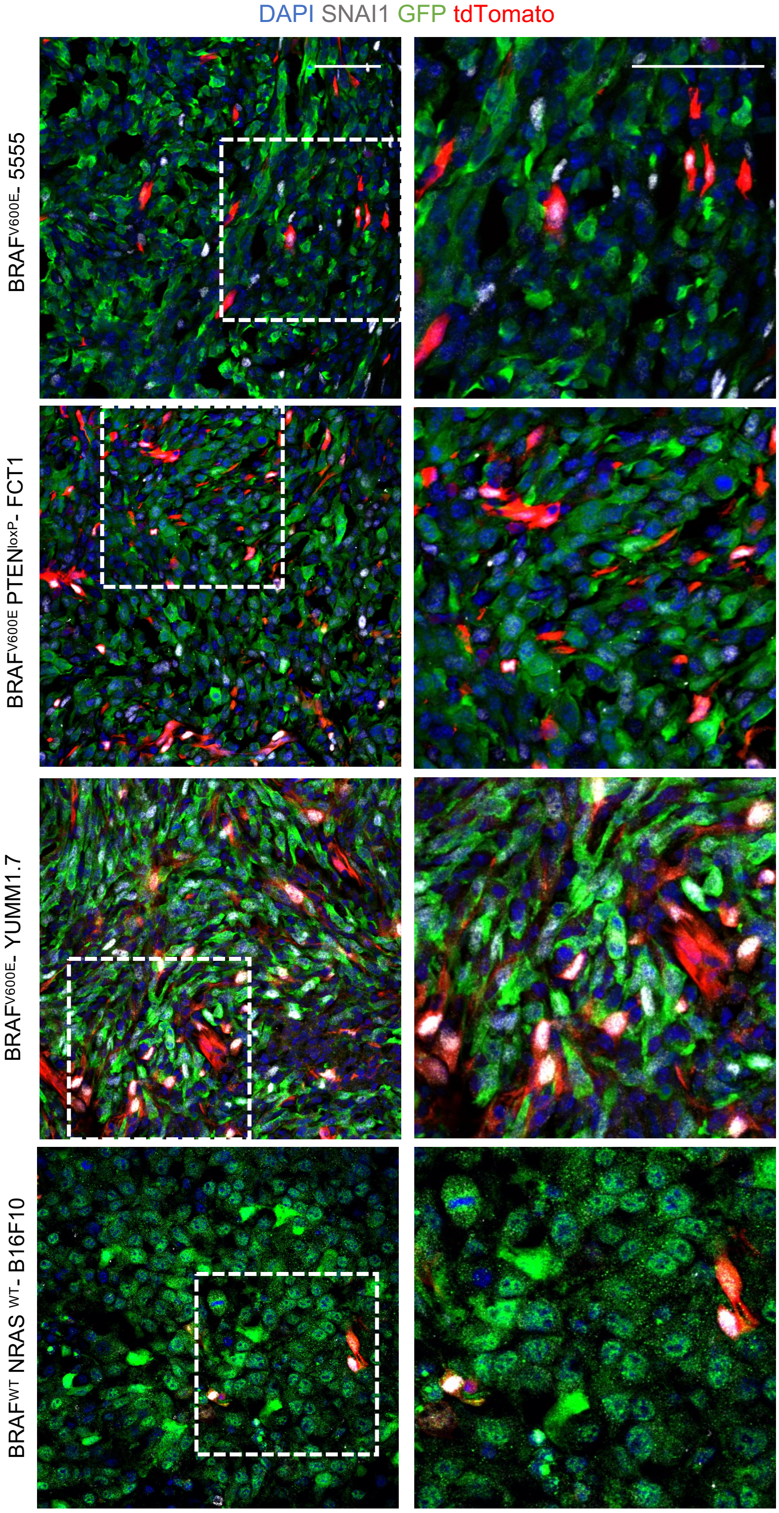

Supplementary Figure 2 (Related to Figure 2)

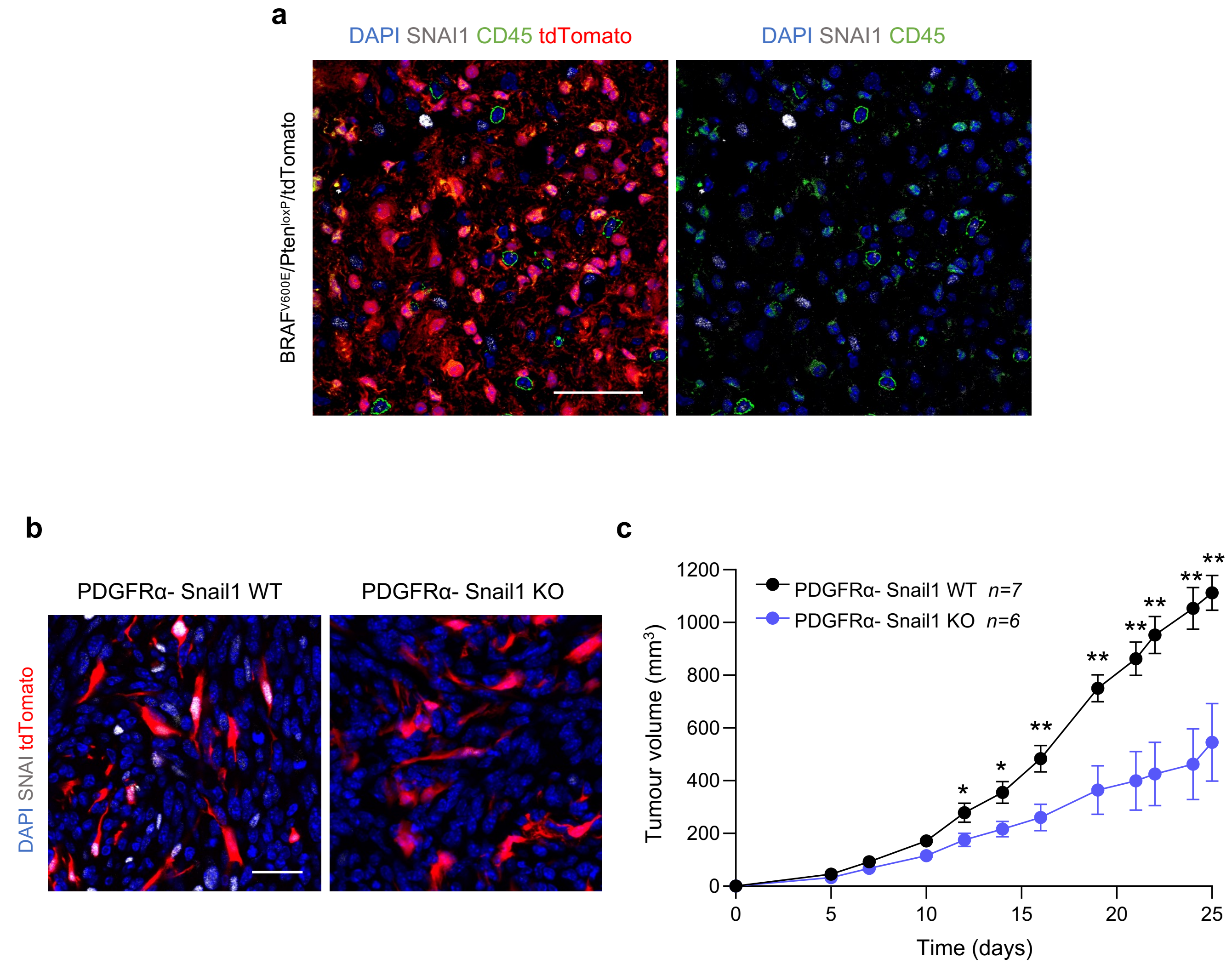

Supplementary Figure 3 (Related to Figure 2)

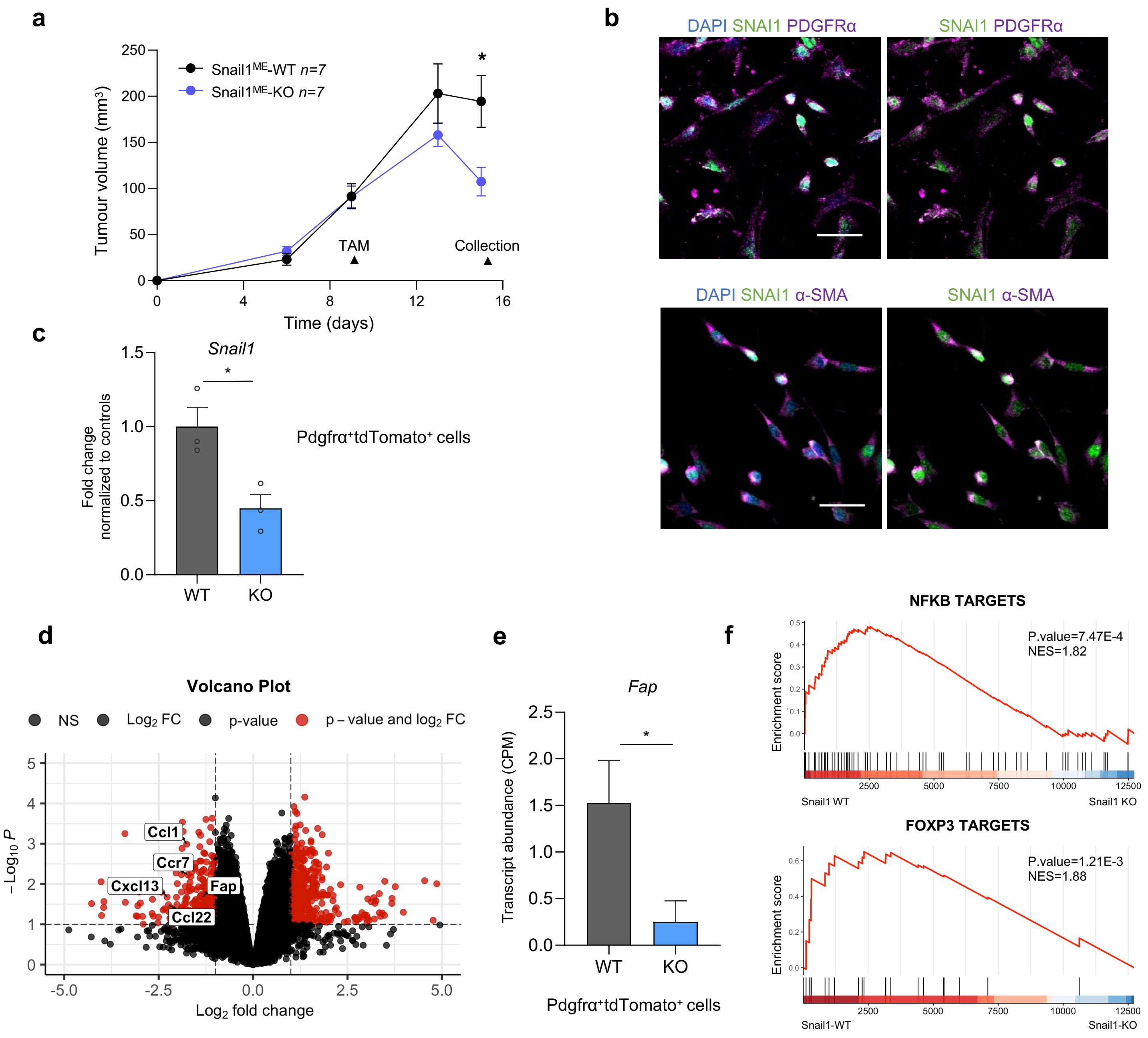

Supplementary Figure 4 (Related to Figure 4)

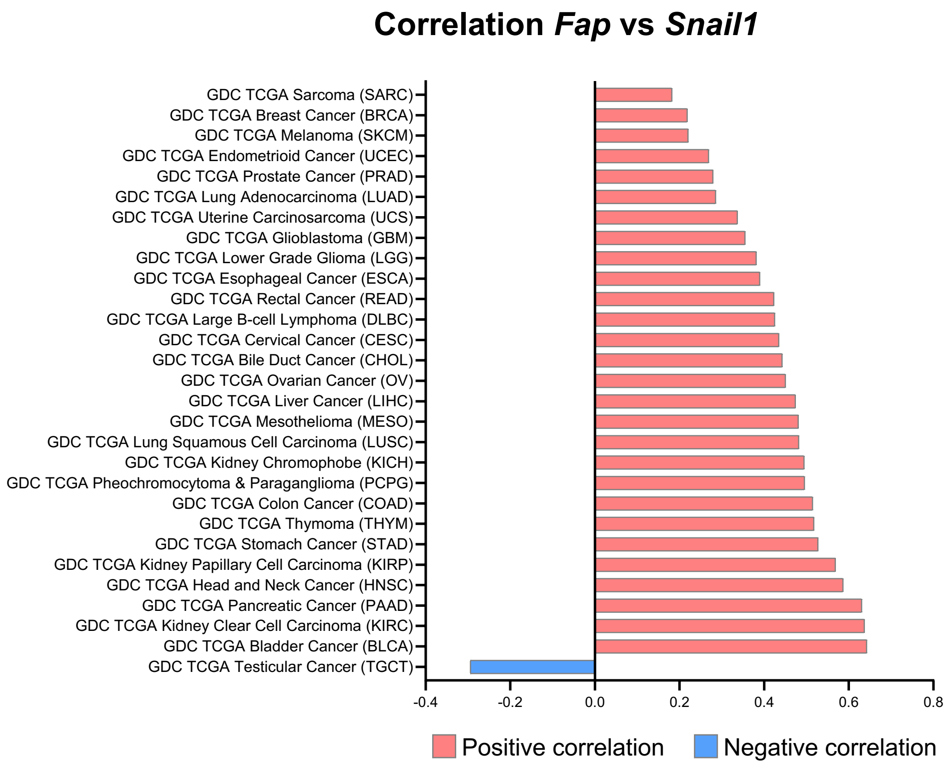

Supplementary Figure 5 (Related to Figure 5)

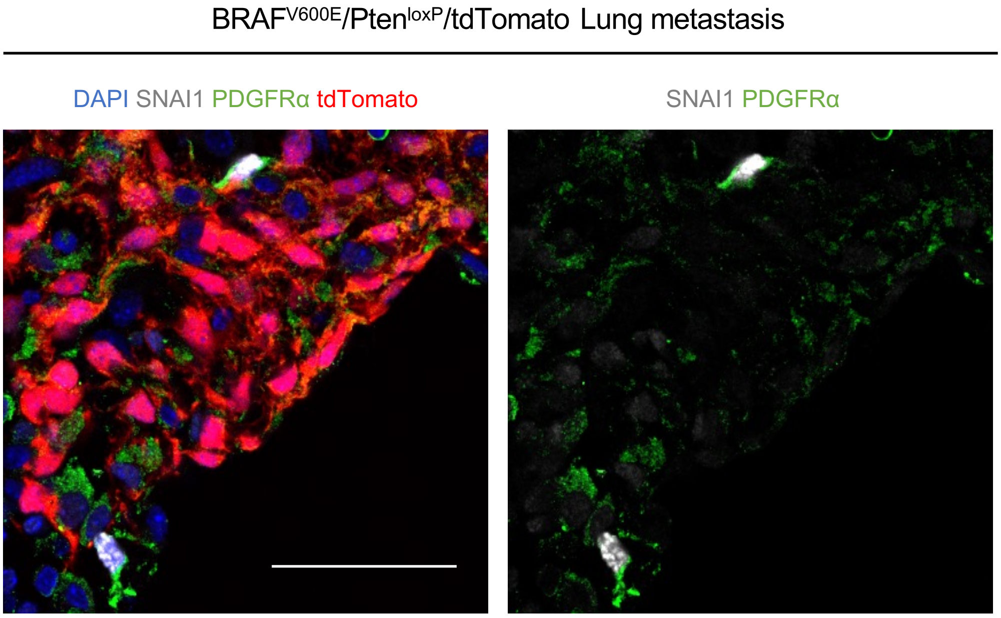

Supplement: Supplementary file 1 — Supplementary Figures [file 41388_2023_2793_MOESM1_ESM.pdf]
