# Supplementary material for: Microenvironmental Snail1-induced immunosuppression promotes melanoma growth
Source: Oncogene. 2023 Jul 29;42(36):2659–72. doi: 10.1038/s41388-023-02793-5 (PMC10473961; doi:10.1038/s41388-023-02793-5)
Supplement: Supplementary file 2 — Supplementary Figure Legends [file 41388_2023_2793_MOESM2_ESM.docx]

**SUPPLEMENTARY FIGURE LEGENDS**

**Supplementary Figure 1 (Related to Figure 1). Snail1 expression in different syngeneic melanoma models.**

Representative images of double immunolabeling for SNAI1 (white) and GFP (green, melanoma cells) in subcutaneous tumours in tamoxifen-treated Snail1^ME^-WT (stromal cells in red) upon injection of different mouse melanoma cell lines tagged with GFP: 5555 (BRAF^V600E^); FCT1 (BRAF^V600E^PTEN^flox/+^); YUMM1.7 (BRAF^V600E^PTEN^flox/flox^Cdkn2a^-/-^); B16F10 (BRAF^WT^NRAS^WT^). Scale bars: 50µm.

**Supplementary Figure 2 (Related to Figure 2). Snail1 targeting in PDGFRα^+^ fibroblasts reduces melanoma growth.**

**(a)** Representative images of double immunolabeling for SNAI1 (white) and CD45 (green, immune cells) in BRAF^V600E^/Pten^loxP^/tdTomato melanoma model. tdTomato indicates melanoma cells (red). **(b)** Representative images of immunolabelling for SNAI1 (white) in PDGFRα-CreERT2-tdTomato (PDGFRα-Snail1 WT) (left) and PDGFRα-CreERT2-tdTomato-Snail1^fl/fl^ (PDGFRα-Snail1 KO) (right) tumours upon tamoxifen administration. tdTomato indicates PDGFRα^+^ cells (red). **(c)** Braf^V600E^-5555 tumour growth curves in PDGFRα-Snail1 WT and PDGFRα-Snail1 KO mice (n=7 WT and n=6 KO). Data are represented by Mean±SEM and statistically significant differences are tested by unpaired two-tailed Student t-test (*=p<0.05 and **=p<0.01). Scale bar: 50µm.

**Supplementary Figure 3 (Related to Figure 2). Isolation strategy and transcriptomic analysis of Pdgfrα^+^ CAFs from Snail1^ME^-WT and Snail1^ME^-KO mice.**

**(a)** *In vivo* experiment designed to isolate fibroblast from Braf^V600E^-5555 melanomas grown subcutaneously in Snail1^ME^-WT and Snail1^ME^-KO mice. Tumour growth graph of an experiment showing the selected time point for the CAFs isolation (Snail1^ME^-WT=7 and Snail1^ME^-KO=7). **(b)** Representative images of double immunolabeling for SNAI1 (green) and different CAFs markers (magenta), in cells isolated from tumours in (a) after FACS-sorting (Pdgfrα^+^tdTomato^+^GFP^-^). Scale bars: 50µm. **(c)** *Snail1* mRNA levels assessed by RT-qPCR to validate the fibroblasts population isolated by FACS (Pdgfrα^+^tdTomato^+^GFP^-^) from tumours in (a) (samples from 3 animals with the same genotype were pooled for each condition). **(d)** Volcano plot of Log_2_ fold change of DEGs between Snail1-WT and Snail1-KO fibroblast samples. The red dots on the right represent the upregulated genes and the red dots on the left the downregulated genes. **(e)** Transcript quantification in Pdgfrα^+^tdTomato^+^ fibroblasts subjected to RNA-Seq (Fig. 2d, e) (counts per million, CPM) from melanomas growing in Snail1-WT (WT) and Snail1-KO (KO) mice. **(f)** Gene set enrichment analysis (GSEA) showing enrichment of the indicated signatures in the Snail1^ME^-WT and Snail1^ME^-KO CAFs from tumours. NES, normalized enrichment score. Data are represented by Mean±SEM and statistically significant differences are tested by unpaired two-tailed Student t-test (*=p<0.05).

**Supplementary Figure 4 (Related to Figure 4). *Fap* and *Snail1* correlation across cancer types.**

Pearson correlation analysis between *Fap* and *Snail1* expression using all cancer types from The Cancer Genome Atlas program (TCGA) database. The graph includes cancer types with a significant correlation (p-value <0.05). Positive correlation is represented in red and negative correlation in blue.

**Supplementary Figure 5 (Related to Figure 5). Snail1 is expressed in Pdgfrα^+^CAFs in lung metastases from an inducible BRAF^V600E^/Pten^loxP^/tdTomato melanoma model.**

Representative images of double immunolabeling for SNAI1 (white) and PDGFRα (green, fibroblasts) in BRAF^V600E^/Pten^loxP^/tdTomato melanoma lung metastases. tdTomato indicates melanoma cells (red). Scale bar: 50µm.
